# Postoperative evaluation of special needs and healthy patients with endodontic treatment under general anesthesia: a retrospective research

**DOI:** 10.1186/s12903-024-04584-0

**Published:** 2024-07-14

**Authors:** Busenaz Cemile Uysal, Hicran Donmez Ozkan, Ozlem Kocaturk

**Affiliations:** 1 Department of Endodontics, Faculty of Dentistry, Aydın Adnan Menderes University, Aydın, Turkey; 2 Department of Oral and Maxillofacial Surgery, Division of Anesthesiology, Faculty of Dentistry, Aydın Adnan Menderes University, Aydın, Turkey

**Keywords:** Postoperative discomfort, General anesthesia, Dental treatment, Patients with special needs

## Abstract

**Background:**

This retrospective clinical study was undertaken to comparatively evaluate the number of restorative treatments, endodontic treatments, and tooth extractions performed for patients under general anesthesia due to dental anxiety or special needs between 2015 and 2022 and to examine the pain, bleeding, nausea, and vomiting data of those patients.

**Methods:**

In total, 1165 patients underwent dental treatment under general anesthesia in the faculty hospital. Those under the age of 15 and with no endodontic procedure planned (*n* = 918) were excluded, followed by those with incomplete data (*n* = 25) and those without endodontic treatment (*n* = 25). Patients who underwent at least one endodontic treatment were finally included in the study (*n* = 184). Patients were divided into two groups: healthy and with special needs. Dental treatments were recorded as endodontic, restorative, and teeth extractions. Endodontic treatments were classified according to the tooth type (premolar, molar, and incisors). The composite restorations were classified as anterior, occlusal (O), occluso-distal (OD) or occluso-mesial (OM), and mesio-occluso-distal (MOD) restorations and patients’ post-treatment pain, nausea, vomiting, and bleeding were recorded. The data were analyzed statistically.

**Results:**

Among the 184 patients included in the study, 70 (38%) were healthy, and 114 (62%) had special needs. Postoperative bleeding was observed more in patients with special needs (χ^2^ = 4.189, *p* < 0.05), whereas pain was observed more in healthy patients (U = 2922.00, *p* < 0.05). While the number of anterior, O, and MOD restorations was higher in patients with special needs, the number of OD or OM restorations was higher in healthy patients (χ^2^ = 74.877, *p* < 0.05).

**Conclusions:**

Patients with special needs undergo a greater number of restorative treatments compared to control patients, which may be associated with the inadequate oral hygiene care of such patients. However, restorative treatment is mostly indicated for such patients in our faculty hospital, which may indicate that a conservative approach is taken. Additionally, the finding that postoperative bleeding was more severe in this group of patients compared to the control group in this study may emphasize the need to consider more possible complications after general anesthesia in these patients.

## Background

Nowadays, most dental treatments are performed under local anesthesia. However, due to the need for invasive intervention and patient-related reasons, outpatient general anesthesia (GA) applications are frequently preferred [1]. Patients who are candidates for GA include those with special mental and/or intellectual needs who have difficulty cooperating, those with anxiety, those with psychological disorders, those with a severe nausea reflex, patients who report that they cannot tolerate the procedure under local anesthesia, those who are rarely allergic to local anesthesia, those who cannot be anesthetized in the relevant area locally due to acute inflammation, and those who cannot tolerate the procedure while awake due to the long duration of the procedure [1].

A spectrum of challenges spanning physical, intellectual, sensory, and mental health domains often hinder the effective dental treatment of patients with special needs. Due to these cooperative or physical challenges, such patients often necessitate dental treatment under GA. Physical limitations such as difficulty in transferring to the dental chair or maintaining balance are prevalent among patients with conditions like spinal cord injuries, cerebral palsy, multiple sclerosis, and muscular dystrophy [2]. Intellectual barriers manifested through communication and comprehension difficulties may hinder patients with conditions like Down syndrome, autism spectrum disorder, and intellectual developmental disorders from fully understanding treatment procedures [3]. Sensory sensitivities, including those related to hearing and vision impairments, can exacerbate discomfort during dental visits [4, 5]. Moreover, patients with mental health conditions like post-traumatic stress disorder (PTSD) or schizophrenia may necessitate additional support and patience to navigate dental care effectively [6].

The demand for dental treatment under GA is constantly growing, especially in patients whose cooperation is not possible, such as young children, people with special mental needs, or people with advanced dental anxiety, as supported by the literature [7−9]. According to the Helsinki Public Dental Service, the main reasons why dental treatments are performed under GA are difficulty in cooperation (65%), dental phobias (37%), and the need for urgent dental treatment (26%) [1]. Jockusch et al. stated that among their four assigned groups (persons with special needs, dementias, dental phobias, and addictions/psychosocial disorders), mostly people with special needs (*n* = 154, 69.7%) receive dental treatment under GA [7].

Dental anxiety, often linked to pain concerns, can lead to avoidance of dental care, resulting in poor oral health [10]. Managing dental anxiety is essential to prevent a cycle of neglect [11] so that patients can receive better care [10]. Specialized anesthesia techniques are vital in managing dental anxiety and improving patient outcomes. Administering local anesthesia remains a common method in dentistry. However, in a study on dental care provided under GA, Savanheimo et al. emphasize the importance of GA in dental procedures [1]. Study on the use of sedation and GA by dentists underscores the significance of these practices in dental care [12]. Behavioral management techniques are also crucial to promoting patient coping and providing appropriate care [13]. It is important to prioritize these noninvasive methods to avoid sedation or GA because of their complication risks [14]. However, if it is difficult to maintain patients’ cooperation due to severe dental anxiety or having special needs, conservative dental care under GA can promote maintaining a functional dentition [15].

Restorations, endodontic treatments, and periodontal and surgical procedures can be performed under GA [1]. Patients with special needs are more prone to oral and dental health problems [15]. They have a higher incidence of caries due to their special diet (such as nasogastric tube and gastronomy feeding), medications, and severe impairment in motor function [15]. Deficiencies in chewing functions and improper brushing habits increase the progression of the problem [15]. Loss of a large number of teeth results in oral dysfunction. The preservation of existing teeth is essential to preventing dysphagia and increasing chewing capacity [15]. The primary goal of clinicians is to maximize the patient’s masticatory function and provide full oral rehabilitation by minimizing other oral health problems [15]. Root canal treatment under GA is indicated for functional teeth that show signs of pulpal necrosis [15]. Although technically difficult and time-consuming, root canal treatment is essential for oral rehabilitation under GA and is increasingly reported [15]. However, all teeth that cannot be restored should be extracted unless there is a contraindication [16].

After the administration of GA, respiratory and cardiovascular complications, such as airway obstruction and hypotension, may occur in the postoperative care unit [17]. Complications due to dental treatment, such as allergic reactions, anaphylaxis, neurological complications, nausea, vomiting, and severe bleeding from extraction sockets, are also possible [17]. A recent study has found a significant relationship between procedures performed and postoperative pain [18]. Effective management of postoperative pain seems to be effective in reducing recovery time and hospital stay. Postoperative pain from tooth extractions performed during GA can be managed by using an intraoperative nerve block or local anesthetic infiltration and prescribing various medications [19]. Postoperative nausea and vomiting (PONV) is one of the most common complications after GA [20] and a common reason for delayed discharge [21]. Nausea and vomiting complications are mostly due to opioid and inhaler anesthetics [22, 23]. However, bleeding and pain depend on the procedure and the patient’s substitution [24, 25]. As anesthesia and surgical procedures have become safer, postoperative complications, such as pain, nausea, and vomiting, have become more prominent [26]. In the literature, there are few studies evaluating postoperative comfort in dental procedures completed under GA [27].

Our study collectively emphasizes the importance of using GA due to a lack of cooperation of patients with special needs and dental anxiety, the postoperative complication management of patients with special needs and dental anxiety, and the improvement of patient outcomes in dental practices. By addressing these aspects, dental professionals can enhance patient care, promote better oral health outcomes, and contribute to the development of best practices in dental anesthesia. In light of all this information, the purpose of this retrospective clinical study was to comparatively evaluate the number of restorative treatments, endodontic treatments, and tooth extractions performed for patients under GA due to dental anxiety or special needs between 2015 and 2022 and to examine the postoperative pain, bleeding, nausea, and vomiting data of the patients.

## Materials and methods

This retrospective study was conducted according to the principles outlined in the Declaration of Helsinki. Ethics committee approval of this study was received from Aydın Adnan Menderes University Faculty of Medicine Non-Interventional Clinical Research Ethics Committee (2022/107). This article has been reported using a statement from STROBE (Strengthening the Reporting of Observational Studies in Epidemiology) as closely as possible. Informed consent was obtained from all the participants enrolled in the present study.

### Patient selection

This study included a total of 184 patients who underwent root canal treatment on at least one tooth under GA due to their severe dental treatment anxiety or special needs at the Faculty of Dentistry Hospital between 2015 and 2022. The inclusion and exclusion criteria of the patients in the study are presented in Table 1.

**Table 1:** Inclusion and exclusion criteria of the patients in the study

Inclusion Criteria	Exclusion Criteria
Having received dental treatment under general anesthesia between January 2015 and August 2022	Patients whose teeth do not have endodontic treatment
Root canal treatment has been performed on at least one tooth of the patient.	Incomplete recording of patient data
Patients over 15 years of age	

The total number of patients who underwent dental treatment under GA was 1165. Out of these patients, patients under the age of 15 who applied to the Pedodontics Department and patients with no endodontic procedure planned or performed were 918. The number of patients who applied to the Endodontics Department was 247. Patients with incomplete data (*n* = 25) and who could not have endodontic treatment (e.g., due to extraction and filling) (*n* = 25) were excluded. The total number of patients included in the study was 184. Then, patients were divided into two main groups: healthy patients who have dental anxiety and patients with special needs.

### Dividing patients into groups

The group of patients with special needs consisted of individuals with autism (*n* = 40), cerebral palsy (*n* = 35), syndromes accompanied by mental retardation (e.g., Down syndrome) (*n* = 52), epilepsy (*n* = 35), cerebrovascular disease sequelae (*n* = 4), hemiparesis (*n* = 7), motor dysfunctions (*n* = 4), bipolar disorder (*n* = 5), and schizophrenia (*n* = 3). The group of healthy patients were ASA 1 patients who had dental anxiety (*n* = 70). This group consisted of patients unable to open their mouths due to dental anxiety, who could not cooperate, and we could not even examine with a dental mirror or probe.

Demographic data of the patients, such as age, weight (kg), and gender, as well as operation duration and recovery time, were examined and recorded. Endodontic treatments were classified according to the type of tooth (premolar, molar, and incisors). The composite restorations were classified as anterior restorations, occlusal restorations (O), occluso-distal (OD) or occluso-mesial (OM) restorations, and mesio-occluso-distal (MOD) restorations.

### Treatments applied to patients

The GA procedures were standardized in all patients. Preoperative fasting was ensured for 8 h before GA was performed. The anesthesiologist followed standard monitoring of all patients during the procedure. Vascular access was established, and standard induction was applied with intravenous (IV) fentanyl (1 mcg/kg), propofol (2.0−2.5 mg/kg), and rocuronium (muscle relaxant) (0.5 mg/kg) for anesthesia induction. After the muscle relaxant effect was established, nasal endotracheal intubation was performed on each patient. Patients are intubated nasally to achieve good visibility of the field and suitable manipulation for dental procedures. Following intubation, anesthesia was maintained with a combination of sevoflurane (2–4 L/min), 50% oxygen, and 50% nitrogen protoxide. An oropharyngeal tampon was placed to prevent the patient from aspirating the materials, residues, and fluids used during the operation. A silicon bite/dental mouth opener was applied to all patients during the routine procedure. Local anesthesia was applied to the area where tooth extraction would be performed with 4% articaine hydrochloride (Ultracain D-S Ampul, Aventis, Istanbul) containing 1:200,000 epinephrine.

Dental treatments performed under GA were restorative procedures (composite and GIS), endodontic treatments (root canal treatments, amputation, and capping), tooth extractions, cyst enucleation, biopsy, and determination and prosthetic procedures (e.g., tooth preparation and post application).

Teeth that were endodontically treated were examined clinically and radiologically. Then, the access cavity of the tooth was prepared, and the root canal orifices were detected. The working length was determined using an electronic apex locator (DTE Dpex III Apex Locator, Woodpecker, Guilin, China) and a #10−#15 K type canal file (Dentsply Maillerfer, Ballaigues, Switzerland) to be 1 mm shorter than the (0.0) value shown by the device. The canals were prepared using the crown-down technique and the ProTaper Next (Dentsply Maillefer) rotary system at the torques and speeds recommended by the manufacturer. After each file, the canals were irrigated with 2 mL of 5.25% NaOCl. After the shaping was completed, the appropriate master cone was selected and confirmed by periapical radiography. The final irrigation protocol was applied with 17% EDTA, 5.25% NaOCl, distilled water, and 2% CHX, respectively. The canals were filled with the lateral compaction method and resin-containing root canal sealer (ADSEAL, Meta Biomed, Korea). Then, they were restored using adhesive procedures.

During the restorative procedures, cavities were prepared with diamond burs. Then, 37% phosphoric acid (Scotchbond Universal Etchant, 3 M, USA) was applied to enamel surfaces for 30 s and to dentin surfaces for 15 s. Following these steps, the cavities were rinsed with water for 30 s and dried with compressed air for 15 s. Adhesive resin (Dentsply Prime & Bond Elect Universal Bond, Dentsply Sirona, Germany) was applied to the dried cavities for 20 s according to the manufacturer’s instructions and polymerized by applying light for 20 s with an LED light device (Elipar FreeLight S10, 3 M ESPE). Posterior composite resin (Estelite Quick Posterior Kompozit, Tokuyama, Japan) was placed in the cavity with a wedge-shaped oblique incremental technique in 2 mm layers following the manufacturer’s recommendation, and each layer was polymerized with an LED light device (1200 mW/cm²) for 20 s. The restorations were completed after polishing. All the other dental treatment needs, such as periodontologic, prosthetic, or oral surgery, have been performed by specialists who are experts in the related area.

Following the end of the dental treatments, the anesthesiologist advised standard post-anesthesia recommendations to the families. Recommendations were provided pertaining to the analgesic medication to be administered post-discharge, instructions regarding the appropriate recourse in the event of complications, designated contacts for assistance, as well as guidance on managing bleeding, nausea, and vomiting. The dentist advised information about the dental procedures performed and oral hygiene. Patients were taken to the recovery unit in the postoperative period and discharged after routine service observations were completed. The type and number of all procedures performed were recorded from patient files on case report forms.

### Postoperative evaluation

#### Postoperative pain evaluation

In the evaluation of pain, the Wong−Baker Facial Scale was used for patients with special mental needs. In cases where the Wong−Baker Facial Scale was insufficient, the FLACC pain scale was used. In the FLACC pain scale, a 0 score indicates that the patient is calm and comfortable, scores between 1−3 indicate that the patient is slightly disturbed, scores between 4 −6 indicate that the patient is in moderate pain, and scores between 7−10 indicate that the patient is significantly disturbed [28].

Patients with anxiety were evaluated with the Numerical Pain Scale (NRS). The NRS has numbers from 0 (no pain) to 10 (unbearable pain) [28].

Postoperative pain levels of patients with anxiety complaints were recorded at 0, 2, and 6 h in the recovery room.

If the patients expressed a pain score of more than 4 on the NRS scale, various analgesics were administered, and their route of administration (IV or intramuscular) and application doses were recorded.

#### Postoperative bleeding and PONV evaluation

Bleeding and PONV conditions of the patients whose treatment was completed under GA were recorded at 0, 2, and 6 h. Two or more retching episodes were considered nausea. The presence of vomiting was also recorded on the forms. The various antiemetics were administered, and their doses were recorded. Bleeding status in the operation areas of the patients was marked as present or absent, and bleeding was considered to be present regardless of whether it was leaking or intense.

### Statistical analysis

The IBM SPSS Statistics version 25 (IBM Corp., Armonk, NY, USA) package program was used for the statistical analysis. For descriptive statistics, the mean, standard deviation, minimum-maximum values, and percentages were used. The Chi-square test was performed to examine the relationship between groups, the Shapiro−Wilk test was used to determine whether the data showed normal distribution, and the Mann−Whitney U test was used to evaluate the variables in two groups. All tests were performed at the 0.05 significance level.

## Results

**Fig. 1:**
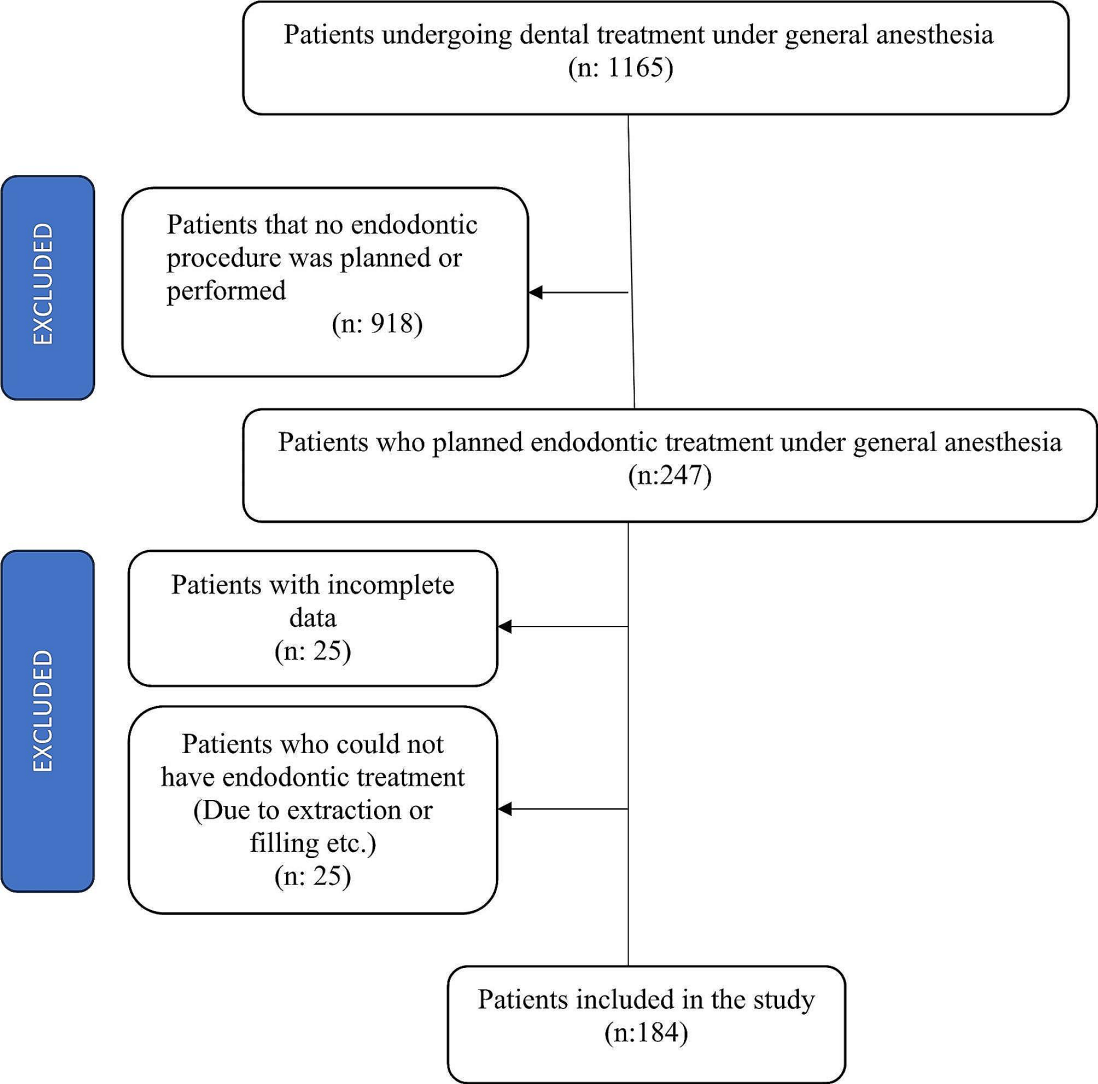
Flow chart of the patients included in the study

A total of 184 patients over the age of 15 who had endodontic treatment on at least one tooth under GA between 2015 and 2022 were included in this study (Fig. 1).

### Demographic data

According to the status of the patients, 38% (*n* = 70) were healthy (had dental anxiety), and 62% (*n* = 114) had special needs. Reasons for patients being referred to GA were autism (*n* = 40), cerebral palsy (*n* = 35), syndromes accompanied by mental retardation (e.g., Down syndrome) (*n* = 52), epilepsy (*n* = 35), cerebrovascular disease sequelae (*n* = 4), hemiparesis (*n* = 7), motor dysfunctions (*n* = 4), bipolar disorder (*n* = 5), schizophrenia (*n* = 3), and dental anxiety (*n* = 70). It was observed that the most common comorbid disease in patients with special needs was epilepsy (*n* = 35), followed by diabetes (*n* = 10) and hypertension (*n* = 8).

When demographic factors were evaluated, the average age of healthy patients was significantly higher than that of patients with special needs (U = 2477.50, *p* < 0.05). Similarly, the weight (kg) of healthy patients was statistically significantly higher than that of patients with special needs (U = 3104.00, *p* < 0.05) (Table 2).

**Table 2:** Examination of patients’ demographic information according to groups

		Healthy			Special Needs			Total			Test Statistics		
		*n*	%	Mean± Std. Deviation	*n*	%	Mean± Std. Deviation	*n*	%	Mean± Std. Deviation	U	*X* ^2^	*p*
**Age**		70	38	32.30 ± 11.34	114	62	25.38 ± 8.46	184	100	28.01 ± 10.20	2477.50		< 0.001
**Weight (kg)**		70	38	68.99 ± 17.25	114	62	62.74 ± 20.94	184	100	65.11 ± 19.80	3104.00		0.011
**Gender**	**Female**	29	35.8		52	64.2		81	38			0.308	0.579
	**Male**	41	39.8		62	60.2		103	62				
**Operation Duration (minutes)**		70	38	218.77 ± 91.57	114	62	211.99 ± 76.46	184	100	214.57 ± 82.36	3947.00		0.902
**Recovery Time (minutes)**		70	38	14.79 ± 3.81	114	62	15.56 ± 8.38	184	100	15.27 ± 7.00	10,502.00		0.492

Test statistics: Chi-square and Mann−Whitney U tests were used

Out of 184 patients, 11 needed further treatment under GA, 10 needed two GA operations, and 1 needed three operations. In 3 of 11 patients, while root canal treatment was performed at the first operation, physicians considered extraction at the second operation. All of these patients had special needs.

### Evaluation of the number of patients with special needs and healthy individuals who received dental treatment

Of the patients who underwent O restoration, 28% (*n* = 26) were healthy and 72% (*n* = 67) had special needs. The number of patients who underwent O posterior composite restoration in the group of patients with special needs was found to be significantly higher than in the group of healthy patients (χ^2^ = 8, 117, *p* < 0.05) (Table 3).

**Table 3:** Number of patients that received treatment

Dental Treatments	Healthy		Special Needs		Total		*X*^2^ / *p*	U / *p*
	*n*	%	*n*	%	*n*	%		
**Occlusal (O) Restorations**	26	28	67	72	93	50.5	8,117 / 0.005	480,00 / 0.001
**Occluso-distal (OD) or Occluso-mesial (OM) Restorations**	49	47,1	55	52,9	104	56.5	8,352 / 0.004	1040,00 / 0.040
**Mesio-occluso-distal (MOD) Restorations**	62	40	93	60	155	84.2	1.597 / 0.206	2836.00 / 0.858
**Anterior Restorations**	45	36.6	78	63.4	123	66.8	0.335 / 0.563	1481.00 / 0.145
**One-Root One-Canal Root Canal Treatment**	42	37.2	71	62.8	113	61.4	0.095 / 0.758	1484.00 / 0.966
**Two-Canal Root Canal Treatment**	13	52	12	48	25	13.6	2.391 / 0.122	73.50 / 0.810
**Three- and Four-Canal Root Canal Treatment**	34	37	58	63	92	50	0.092 / 0.761	937.00 / 0.588
**Extraction**	55	37.4	92	62.6	147	79.9	0.123 / 0.726	2187.00 / 0.164

Test statistics: Chi-square and Mann−Whitney U tests were used

Among the patients who underwent OD or OM posterior composite restoration, 47.1% (*n* = 49) were healthy, and 52.9% (*n* = 55) had special needs. Regarding the treatments, the number of patients who underwent OD or OM posterior composite restoration was significantly higher in the healthy group compared to the group with special needs (χ^2^ = 8.352, *p* < 0.05) (Table 3). There was no significant difference between the groups in terms of the number of patients who underwent MOD posterior restoration and anterior aesthetic restoration (χ^2^ = 1.59, *p* > 0.05) (U = 1481.00, *p* > 0.05) (χ^2^ = 0.335, *p* > 0 0.05) (Table 3). No significant difference was observed between the groups in terms of the number of patients who underwent root canal treatment (χ^2^ = 0.095, *p* > 0.05), (χ^2^ = 2.391, *p* > 0.05), (χ^2^ = 0.092, *p* > 0.05) (Table 3). There was no significant difference between the groups in terms of the number of patients who underwent tooth extraction (χ^2^ = 0.123, *p* > 0.05) (Table 3).

### Evaluation of the number of endodontic treatments applied to patients

There was no significant difference between the two groups of patients in terms of endodontically treated tooth type (χ^2^ = 2.170, *p* > 0.05) (Table 4).

**Table 4:** Number of endodontic treatments

Endodontic Treatments		Healthy	Special Needs	Total	
**Treatments**	**Incisors** n / %	55/ (39.0%)	85 / (42.1%)	140 (40.8%)	
	**Premolars** n / %	42 / (29.8%)	46 / (22.8%)	88 (25.7%)	
	**Molars** n / %	44 / (31.2%)	71 / (35.1%)	115 (33.5%)	
**Total**		141/ (41.1%)	202 / (58.9%)	343 (100%)	
**χ²** ***/ p***		3,080 /0,214	1,410 / 0.494	**2**,**170 / 0.338**	

Test statistics: Chi-square tests were used

### Evaluation of the number of restorative treatments applied to patients

A significant difference was found between the groups in terms of the number of restorative treatments (χ^2^ = 74.877, *p* < 0.05). According to this, the distribution of the number of procedures for all restorative procedures is different according to the groups. While anterior aesthetic restoration, O restoration, and MOD restoration were more common in patients with special needs, OD or OM restorations were more numerous in healthy patients. The largest difference in terms of procedures between the two groups of patients was observed in patients with an O restoration application. This was followed by anterior restoration (Table 5).

**Table 5:** Number of restorative treatments

Restorative Treatments		Healthy	Special Needs	Total
**Treatments**	**Anterior Restoration** n / %	139 / (29.0%)	292 / (34.5%)	431 / (32.5%)
	**Occluso (O) Restoration** n / %	45 /(9.4%)	202 /(23,8%)	247 / (18.6%)
	**Occluso-distal (OD) and/or Occluso-mesial (OM) Restoration** n / %	144 / (30.0%)	125 / (14.8%)	269 / (20.3%)
	**Mesiao-occluso-distal (MOD) Restoration** n / %	152 / (31.7%)	228 / (26.9%)	380 / (28.6%)
**Total**		480 / (36.2%)	847 / (63.8%)	1327 / (100%)
**χ** ^**2**^ **/** ***p***		4,098 / 0.251	10,255 / 0.017	74,877 / <0.001

Test statistics: Chi-square tests were used

### Evaluation of the number of surgical treatments applied to patients

There was no significant difference between the patient groups in terms of the number of teeth extracted (χ^2^ = 1.387, *p* > 0.05) (Table 6).

**Table 6:** Number of tooth extractions

Tooth Extraction		Healthy	Special Needs	Total	
**Treatments**	**Incisors n / %**	39 / (18.7%)	60 / (17.2%)	99 (17.8%)	
	**Premolars n / %**	31 / (14.8%)	65 / (18.7%)	96 (17.2%)	
	**Molars n / %**	139 / (66.5%)	223 / (64.1%)	362 (65.0%)	
Total		**209 / (37.5%)**	348 / (62.5%)	557 (100%)	
χ^2^ / *p*		**6**,**397 / 0.041**	**12**,**216 / 0.002**	**1**,**387 / 0.500**	

Test statistics: Chi-square tests were used

### Evaluation of patient complications

Comparing the two patient groups, postoperative bleeding was significantly more common in patients with special needs (χ^2^ = 4.189, *p* < 0.05); however, there was no significant difference in terms of postoperative pain (χ^2^ = 3.040, *p* > 0.05) and PONV (χ^2^ = 0.633, *p* > 0.05) (Table 7).

**Table 7:** Distribution of patient groups included according to complications

Complications	Healthy *n* / %	Special Needs *n* / %	Total	χ^2^ / *p*
Postoperative Bleeding	9 / (23.7%)	29 / (76.3%)	38 / (20.%)	4.189 / 0.041
Postoperative Pain	46 / (43.4%)	60 / (56.6%)	106 / (57.6%)	3.040 / 0.081
PONV	20 / (33.9%)	39 / (66.1%)	59 / (32.1%)	0.633 / 0.426
No Complications	12 / (32.4%)	25 / (67.6%)	37 / (20.1%)	0.659 / 0.432

Test statistics: Chi-square tests were used

Postoperative bleeding occurred after endodontic and restorative treatment in a total of 38 patients; 23.68% (*n* = 9) of these patients were healthy, and 76.32% (*n* = 29) had special needs. Postoperative bleeding occurred after the surgical procedure in a total of 33 patients; 21.21% (*n* = 7) of these individuals were healthy, and 78.79% (*n* = 26) had special needs. Postoperative pain occurred after endodontic and restorative procedures in a total of 106 patients. Postoperative pain occurred after the surgical procedure in a total of 93 patients. PONV occurred in a total of 59 patients after endodontic and restorative procedures. PONV occurred in a total of 43 patients after the surgical procedure (Table 8).

**Table 8:** Distribution of procedures applied to patients according to complications

	Postoperative Bleeding			Postoperative Pain			PONV			
	Healthy	Special Needs	Total	Healthy	Special Needs	Total	Healthy	Special Needs	Total	
**Endodontic** **and Restorative Treatments** **n (%)**	9 (23.68%)	29 (76.32%)	38 (100%)	46 (43.40%)	60 (56.60%)	106 (100%)	20 (33.90%)	39 (66.10%)	59 (100%)	
**Extraction** **n (%)**	7 (21.21%)	26 (78.79%)	33 (100%)	39 (41.94%)	54 (58.06%)	93 (100%)	15 (34.88%)	28 (65.12%)	43 (100%)	

Test statistics: Chi-square tests were used. PONV: Postoperative nausea-vomiting

### NRS scores

There was a significant difference between the two groups of patients in terms of NRS 0 h mean values (U = 2922.00, *p* < 0.05), and the NRS 0 h pain score was relatively higher in healthy patients (Table 9).

**Table 9:** Distribution of NRS scores among patient groups

NRS Scores	Healthy	Special Needs	Total	U / *p*
**NRS 0 h** Mean ± Std. Deviation (Min-Max)	2.76 **±** 2.52 (0−9)	1.54 **±** 2.01 (0−8)	2.01 **±** 2.29 (0−9)	**2922.00 / 0.001**
**NRS 2 h** Mean ± Std. Deviation (Min-Max)	0.59 **±** 1.28 (0−6)	0.41 **±** 1.1 (0−5)	0.48 **±** 1.17 (0−6)	3644.00 / 0.145
**NRS 6 h** Mean ± Std. Deviation (Min-Max)	0.16 **±** 0.69 (0−5)	0.07 **±** 0.393 (0−3)	0.1 **±** 0.528 (0−5)	3846.00 / 0.272

Test statistics: Mann−Whitney U tests were used

### Analgesic and antiemetic use

Analgesic use was significantly higher in healthy patients than in those with special needs (χ^2^ = 6.906, *p* < 0.05) (Table 10). The most commonly used analgesic was diclofenac sodium (Table 11). No significant difference was found between the two groups of patients in terms of antiemetic drug use (χ^2^ = 3.208, *p* > 0.05) (Table 12).

**Table 10:** Analgesic use rate among patient groups

	Healthy		Special Needs		Total	
	*n*	%	*n*	%	*n*	%
**Analgesic Use** **(-/+) (%)**	42/28	(60.00%)/(40.00%)	89/25	(78.10%)/(21.90%)	131/53	(71.20%)/(28.80%)
**χ** ^**2**^	6.906					
***p***	**0.009**					

Test statistics: Chi-square tests were used

**Table 11:** Types of analgesics used by patients

	Healthy	Special Needs	Total
	**n / %**	**n / %**	**n / %**
Diclofenac Sodium	15 / 53.60%	12 / 48.00%	27 / 50.90%
Midazolam	0 / 0.00%	4 / 16.00%	4 / 7.50%
Tenoxicam	5 / 17.90%	0 / 0.00%	5 / 9.40%
Paracetamol	5 / 17.90%	2 / 8.00%	7 / 13.20%
Fentanyl	2 / 7.10%	5 / 20.00%	7 / 13.20%
Tramadol Hydrochloride	1 / 3.60%	2 / 8.00%	3 / 5.70%
Total	28 / 52.80%	25 / 47.20%	53 / 100.00%

**Table 12:** Antiemetic use rate among patient groups

	Healthy		Special Needs		Total		
	**n**	%	**n**	%		**n**	%
**Antiemetic Use** **(-/+) (%)**	56/14	(80.00%) /(20.00%)	102/12	(89.50%)/(10.50%)		158/26	(85.90%)/(14.10%)
**χ** ^**2**^	3.208						
**p**	0.073						

Test statistics: Chi-square tests were used

## Discussion

The primary indications for dental treatments under GA are dental anxiety, severe nausea-vomiting reflex, and lack of cooperation of the patient due to mental or other motor dysfunctions, according to a systemic review based on American clinical guidelines [29]. Consistent with the current literature [1, 29], in our study, the reasons for referring to GA were autism, cerebral palsy, syndromes accompanied by mental retardation (e.g., Down syndrome), epilepsy, cerebrovascular disease sequelae, hemiparesis, motor dysfunctions, bipolar disorder, schizophrenia, and dental anxiety.

Jockusch et al. reported that 69.7% of patients treated under GA had special needs [13]. Similarly, in our study, 62% (*n* = 114) of patients treated under GA had special needs. This prevalence can be attributed to their increased treatment requirements due to challenges in maintaining oral hygiene [14] and the limited availability of dental hospitals providing treatment under GA in the region. As a result of the large number of referred patients, a significant portion of our study population comprised individuals with special needs. While cooperative behavioral techniques or medications may suffice for healthy patients requiring GA [15], those with communication difficulties or special physical needs often necessitate treatment under GA due to the inability to be managed in a traditional dental chair.

In their meta-analysis, Mallineni et al. found no significant difference in the operation time of dental procedures under GA across various studies [14]. Tsai et al. reported operation times ranging from 2.6 to 3.1 h [16]. Our study, similar to those cited, administered comparable treatments to both patients with special needs and healthy patients, with similar time allocations (average 3.57 ± 1.37 h), revealing no disparity between the groups. This uniform approach to healthcare delivery and dental treatment planning is driven by our commitment to equitable service provision. Conversely, some literature suggests that healthy patients may require longer treatment periods and more procedures compared to those with special needs [17].

In this study, a total of 1327 restorative procedures were performed, and the number of restorative procedures performed in patients with special needs was higher than that in the healthy group. The number of endodontic treatments performed was 343, and the number of tooth extractions was 557. No significant differences in these aspects were observed between the two groups of patients. Similarly, Demir et al. stated that the most preferred treatment in healthy individuals is restorative treatment, with an average of 6.8 ± 2.9 treatments per patient, followed by tooth extraction (2.75 ± 2.32) and root canal treatment (1.80 ± 1.56) [30]. According to the findings of our retrospective study, restorative procedures were performed most in both groups, which is in line with the literature [30, 31]. Similar to the literature, the prevalence of total restorative treatments in patients with special needs is higher than that in healthy patients. It is thought that these patients have more dental diseases as a result of challenges in maintaining oral hygiene, their diet, malocclusions, and the teeth becoming a focus of infection due to the lack of chewing functions [32, 33]. The increased need for restorative treatments in patients with special needs could be linked to their compromised oral health status, which may require more extensive interventions [34]. Glassman et al. stated that people with special needs had more dental disease and more missing teeth than the general population and had more difficulty accessing dental care [35]. Low expectations of dental success, fear of treatment, and lack of awareness from caregivers are key factors in problems with access to dental services [36]. A study by Mitsea et al. found that patients with special needs received more dental treatment than healthy patients [37]. As a result, there is a high risk of developing new caries due to a lack of oral hygiene and self-care. A study in the literature reveals that the need for oral care for people with mental special needs cannot be met [38]. Causes such as deficiency in motor functions, dietary patterns, deficiency of chewing functions [33], and disturbances in brushing habits can increase the rate of progression of the problem and create a focus on non-functional teeth infection.

In Jokusch et al.‘s study examining dental treatments of patients with special needs performed under GA, the results of 52% restorative (*n* = 442) and 45.8% surgical (*n* = 389) procedures [39] were found to be similar to our study. However, unlike our study, fewer endodontic treatments (*n* = 19, 2.2%) were performed in that study. In the same study, it was observed that root canal treatments were applied predominantly in the maxillary and mandibular anterior regions. This shows that, unlike our study, operators avoid complex treatments and keep the anesthesia duration as short as possible.

More radical treatments are implemented for patients with special needs, such as cerebral palsy, who do not have a chewing function but only have a swallowing function and/or are fed with nutritional solutions through a nasogastric tube. In these patients, less complex procedures are preferred compared to healthy individuals in order to eliminate complications or the need for repeated treatment [40]. It is thought that tooth extraction under GA may be appropriate as these patients need a more radical approach [9, 39]. Contrary to this situation, in our study, the total number of restorative and endodontic treatments was higher than the number of extractions in both groups, and there was no significant difference between the patient groups receiving endodontic treatment. This shows that we have a more conservative approach to treatments performed under GA. The fact that there is no significant difference between endodontic treatments and extractions performed on patients with special needs and healthy patients shows that we provide equal treatment opportunities to both patient groups in our faculty.

One of the complications that may occur in dental treatments performed under GA is postoperative dental bleeding. Similar to our study, Brailo et al. stated that 36.4% of patients treated under GA experienced postoperative bleeding [27]. Cantekin et al., contrary to our study, reported that the most common postoperative symptom was bleeding, with a rate of 59%, and postoperative dental bleeding was observed in 79.3% of these patients [41]. In our study, postoperative dental bleeding was significantly more common in patients with special needs compared to the healthy group. The reasons for this can be explained by the fact that there are many abnormalities regarding blood count and bleeding profile in individuals with Down syndrome and cerebral palsy [24, 25] and that some epilepsy drugs cause thrombocytopenia [42]. Individuals with trisomy 13 and trisomy 18 and Down syndrome have typical hematological characteristics, such as an increase in the number of circulating nucleic red cells along with mild-transitory neutropenia and thrombocytopenia. Blood counts in individuals with Down syndrome may be normal; the only abnormality may be the dysplastic characteristics of white cells, platelets, and/or red cells [25]. The prevalence of periodontitis in individuals with Down syndrome is higher than observed in the general population and other groups with special needs [43]. These patients are more prone to inflammation and bleeding in the gums than other groups of patients due to the increased prevalence of periodontitis. Patients with cerebral palsy had higher concentrations of antibodies against antithrombin III, a translational product of factor V Leiden mutation, and C and S proteins than healthy patients. Although these clotting abnormalities are important in the etiology of cerebral palsy, they indicate that patients with cerebral palsy are prone to bleeding [24]. Some epileptic drugs also have adverse effects on the clotting system. Carbamazepine, phenytoin, and valproic acid can cause thrombocytopenia. In addition, valproic acid and gabapentin have been associated with acquired von Willebrand disease type 1, hypofibrinogenemia, reduced factor XIII, and abnormal thrombocyte function [42]. If the prognoses of endodontic or restorative treatments performed under GA in patients with special needs are uncertain and doubtful, it is thought that tooth extraction should be preferred [9, 40]. In our study, although there was no significant difference, 62.6% (*n* = 92) of the patients who underwent tooth extraction had special needs, and 37.4% (*n* = 55) were healthy. This difference may have had an impact on postoperative dental bleeding.

In our study, similar to the study by Demir et al. [44], the most common complication was postoperative pain. Postoperative pain occurred in 57.6% (*n* = 106) of 184 patients. Although the 100 mm Visual Analog Scale (VAS) and the 11-point NRS are the most commonly used pain scales in health care, the FLACC pain scale is used in patients who are difficult to communicate with [28]. In our study, the fact that the NRS 0 h score was higher in healthy individuals and the need for more analgesic use may indicate that patients with special needs have difficulty communicating by conventional methods and the inadequacy of the FLACC scale used. Healthy patients experiencing more pain postoperatively could be due to individual pain perception differences, anxiety levels, or variations in pain management strategies [45]. Pain is a subjective phenomenon that varies from person to person, so the golden standard for assessing pain is the person’s own statement [46, 47]. Therefore, clinical evaluation of pain depends on the patient’s ability to convey their experience. In some groups of patients, conditions that prevent the patient from communicating can prevent pain assessment and pain intervention [48]. Although there are studies in the literature regarding the use of the pain scale that was used in this study in patients with special needs [49], the result of our study shows that studies on a larger population are needed.

Cantekin et al. showed in their study that postoperative pain status was related to the number of extractions under GA [41]. Patients who had four or more teeth extracted were more likely to experience postoperative pain. Unlike our study, more patients experienced pain after endodontic and restorative treatments (*n* = 106) than after extraction (*n* = 93). Tooth extractions performed under GA are performed with the support of local anesthesia. The observation in our study that proportionately more extraction procedures were implemented for patients with special needs may have caused them to feel less postoperative pain due to the application of local anesthesia. Hu et al. reported that the rate of postoperative pain was lower in children who underwent tooth extraction under GA (26%), and this may be related to the application of local anesthesia to the area before extraction [50]. Atan et al. have observed patients who undergo dental treatment under GA that the likelihood of experiencing pain in the operation area is reduced with local anesthesia [51]. For these reasons, supporting local anesthesia in endodontic/dental procedures is recommended for postoperative analgesia, even if under GA.

In our study, postoperative pain occurred after endodontic and restorative procedures in a total of 106 patients. Pain associated with endodontics is related to age and gender, root canal morphology, the level of shaping and filling relative to the apical foramen, leaving residual pulp tissue, apical extrusion of irrigating agents and debris, the pathological status of the pulp, previous pain complaints, and OCC trauma [52]. Management of postoperative pain after root canal treatment is multifactorial and is related to the application of good endodontic treatment and appropriate analgesics. Özkan et al. showed that parenterally administered tenoxicam provided more effective analgesia compared to paracetamol in terms of postoperative pain management in root canal treatments completed under GA [53]. In our study, similar to the analgesic drugs mentioned above, it has been observed that both opioids and NSAIDs can be used according to the patient’s needs in postoperative dental pain management. Although there are studies in the literature about pain after endodontic treatment [52], there are very few studies on the evaluation of postoperative pain in patients who received dental treatment under GA [41, 44, 53].

The integration of local anesthesia with GA in dental practices, particularly for endodontic treatments in patients with special needs, dental anxiety, or challenging behaviors, can lead to improved pain management, treatment outcomes, and patient comfort. Incorporating local anesthesia alongside GA can enhance pain control during dental procedures, including endodontic treatments [50]. Moreover, the use of local anesthesia in endodontic procedures is crucial for providing intraoperative analgesia and anesthesia, contributing to the overall success of the treatment [54]. Research has also shown that endodontic treatment under GA can lead to improved treatment conditions and outcomes and increased patient acceptance, particularly in patients with special mental needs [55]. The duration of endodontic treatment under GA can vary based on case complexity, emphasizing the need to accurately assess case difficulty to predict treatment duration [56]. It is important to look for a cost-effective treatment because it is not advised to increase operation time with complex treatments to poor prognosis teeth. The longer the GA becomes, the more complications can occur [57]. The demand for endodontic and other dental treatments under GA for patients with special needs is increasing, highlighting the importance of this approach in providing necessary care to this population [9]. Efforts should be made to encourage patients with special needs to seek dental care earlier to receive primary preventive interventions [58]. Addressing patient barriers, such as cost and quality of care, can help increase the utilization of tooth-retaining procedures, benefiting patients in need of restorative and endodontic treatments [59]. Dental education plays a crucial role in improving access to care for patients with special needs, who are often considered an underserved group in dentistry [60].

All oral surgical interventions (e.g., tonsillectomy and maxillofacial surgery) increase the risk of PONV. According to this study, there is a similar incidence of PONV for all dental procedures performed under GA. It has shown us that all dental procedures, whether invasive or noninvasive, have the same importance in terms of antiemetic prophylaxis [61].

According to a study conducted by Enever et al., the most common postoperative complications after treatment under GA in patients with special needs and children are nausea and vomiting [20]. Enever et al. [20] reported PONV at a rate of 18% in patients with special needs and 21% in healthy patients, which was similar to the 32.1% (*n* = 59) in our study, and there was 20% antiemetic use (*n* = 14), which was also similar to that in our study.

In the meta-analytic review study of drugs used for PONV, Weibel et al. reported that granisetron, dexamethasone, ondansetron, and droperidol showed clinical benefit [62]. Kocatürk et al. stated that PONV was reduced when IV paracetamol was used in the early postoperative period after maxillofacial surgery under GA [63]. In our study, antiemetics were used in line with the literature [62, 63]; metoclopramide and ondansetron were used.

### Limitations

This study design is a retrospective cohort study. Retrospective studies are designed to analyze pre-existing data and thus are subject to biases. The patients who participated in our research included only those who applied to our institution (Faculty of Dentistry, Department of Endodontics/Single Center). Therefore, the results of our study could not reflect the sociodemographic characteristics of patients applying to different centers. Because there are few studies in the literature evaluating patients undergoing endodontic treatment under GA, the results need to be supported by studies with larger patient groups. Inhalation agents, IV anesthetics, and medications used regularly by patients may affect postoperative complications. By including these data, it is possible to conduct prospective studies of different scopes.

## Conclusion

The high number of restorative treatments in patients with special needs can represent the deficiencies of the oral hygiene needs of these patients. The fact that there is no significant difference in terms of endodontic treatments and extractions and surgery time for healthy patients and patients with special needs represents that we provide equal treatment opportunities regardless of the health status of patient groups.

## Data Availability

The datasets generated and/or analyzed during the current study are not publicly available due to ethics approval but are available from the corresponding author upon reasonable request.

## Abbreviations

GA: General anesthesia
FLACC: Face, Legs, Activity, Cry, Consolability
IV: Intravenous
MOD: Mesio-occluso-distal
O: Occlusal
OD: Occluso-distal
OM: Occluso-mesial
PONV: Postoperative nausea and vomiting
NRS: Numerical Rating Scale
